# Faith and vaccination: a scoping review of the relationships between religious beliefs and vaccine hesitancy

**DOI:** 10.1186/s12889-024-18873-4

**Published:** 2024-07-06

**Authors:** Muhammad Haaris Tiwana, Julia Smith

**Affiliations:** Faculty of Health Sciences, Simon Fraser University, Blusson Hall, Room 11810, 8888 University Drive, Burnaby, BC V5A 1S6 Canada

**Keywords:** Vaccine hesitancy, Religion, Sociodemographic, Public health

## Abstract

**Background:**

Throughout history, vaccines have proven effective in addressing and preventing widespread outbreaks, leading to a decrease in the spread and fatality rates of infectious diseases. In a time where vaccine hesitancy poses a significant challenge to public health, it is important to identify the intricate interplay of factors exemplified at the individual and societal levels which influence vaccination behaviours. Through this analysis, we aim to shed new light on the dynamics of vaccine hesitancy among religious groups, contributing to the broader effort to promote vaccine uptake, dispel misunderstandings, and encourage constructive dialogue with these groups.

**Methods:**

We used the PRISMA-ScR (Preferred Reporting Items for Systematic reviews and Meta-Analyses extension for Scoping Reviews) using the 20-point checklist to guide this review. The inclusion criteria for our study were that the literature should be in English, concerned with vaccine hesitancy as the focus of study, study the impact religiosity or religious beliefs as either an outcome or control variable, concerning population levels, and be peer-reviewed.

**Results:**

We analysed 14 peer-reviewed articles that included components related to religiosity or religious beliefs and their impact on vaccine hesitancy published until September 2023. All the articles were published in approximately the last decade between 2012 and 2023, with only 4 of the articles published before 2020. Out of the 14 studies included in our review, twelve utilized quantitative methods, while the remaining two employed qualitative approaches. Among the studies included in our analysis, we found various approaches to categorizing religious belief and identity. In most studies when religion is uniformly regarded as the sole determinant of vaccine hesitancy, it consistently emerges as a significant factor in contributing to vaccine hesitancy. All studies in our review reported sociodemographic factors to some degree related to vaccine hesitancy within their sample populations. Our analysis underscored the need for nuanced approaches to addressing vaccine hesitancy among religious groups.

**Conclusion:**

Vaccine hesitancy is a complex issue and driven by a myriad of individual and societal factors among which religious beliefs is commonly associated to be a driver of higher levels among populations.

**Supplementary Information:**

The online version contains supplementary material available at 10.1186/s12889-024-18873-4.

## Background

Over the past few years, discussions surrounding the acceptance and intention to get vaccinated have grown progressively intricate. The World Health Organization’s Strategic Advisory Group of Experts on Immunization (SAGE) Working Group has described ‘vaccine hesitancy’ as the postponement or outright refusal of vaccination despite the availability of vaccination services [1]. However, this definition has been subject to debate, with a more recent review suggesting that vaccine hesitancy represents a ‘state of indecisiveness’ [2]. In 2019, it was listed among the top 10 global public health threats [3]. In the context of the recent global COVID-19 pandemic, understanding the factors and determinants that influence vaccine hesitancy has become increasingly important, as public health agencies around the globe strive to convince their population to participate in vaccination programs [4]. Vaccine hesitancy exhibits a multifaceted and ever-changing nature across different contexts and periods [5]. Recent research has increasingly explored the social factors contributing to vaccine hesitancy, extending beyond traditional demographics such as age, income, and education. However, there is still ongoing need for further investigation into other determinants such as religion, culture, LGBTQ + identity, disability, and more.

Throughout history, vaccines have proven effective in addressing and preventing widespread outbreaks, leading to a decrease in the spread and fatality rates of infectious diseases, but have also been opposed for various reasons. It is essential to differentiate between vaccine hesitancy and vaccine refusal [6]. Vaccine refusing individuals often hold deeply rooted ideologies influenced by political and cultural views, and create insular communities both in physical and online spaces, which are highly resistant to change [6, 7]. Vaccine hesitancy, on the other hand, is shaped by diverse and interconnected factors, stemming from individual-level fears or concerns like insufficient knowledge, anxiety, or misinformation to more systemic issues in terms of distrust in medical or public systems [6, 8]. Common vaccine hesitant arguments involve concerns that vaccine side effects surpass the diseases they prevent, claims of toxins or disease-causing instances, concerns about mandatory vaccinations violating civil liberties or religious beliefs, and suspicions of doctor bias or pharmaceutical conspiracies [9]. Despite mass media coverage reporting to the contrary, vaccine refusal is much less common than vaccine hesitancy [8].

Public health researchers have sought to understand vaccine acceptance, hesitancy, and rejection mostly as an individualised health problem rather than a collective problem. With some noted exceptions, few conceptualise the underlying social determinants that inform vaccine decision making [10]. Data on COVID-19 vaccinations in the US illustrate trends of vaccine disparities across racialized populations: among the approximate 76% of adult population in the United States that had received one dose, 60.3% were identified as White [11]. In a study conducted in Nigeria, it was discovered that factors like maternal availability and lack of knowledge had the greatest impact on partial immunization, while parental disapproval played a more significant role in cases of non-immunization [12]. Another study conducted on the Measles-Mumps Rubella (MMR) vaccine in the United Kingdom revealed that distinct factors such as level of education, socioeconomic status, safety concerns (particularly in response to fraudulent studies linking MMR vaccines and autism), and social desirability influenced decision-making at each dosage, and the degree of influence also varied at each administration [13].

Considering another layer of complexity is religiosity or religious beliefs. Religious concerns about vaccinations have a long history dating back to hesitations about the smallpox vaccines [14]. As such, religious related convictions are perceived to be among the commonest example for this reticence [15]. Religious faith serves as a significant societal and environmental factor capable of shaping a person’s convictions, their customs, and their health-related actions [16]. The review by Grabenstein provided a detailed elaboration of religious beliefs related to vaccines. For instance, Buddhism and Judaism have no doctrine against vaccines and so more accepting while other’s such as Islam and Christianity are seen as cautious [17, 18]. For instance, perception of porcine ingredients content in polio vaccines was the main barrier identified in Muslim populations. The use of aborted fetal cells and faith in divine protection are concerns raised by members of Amish, Catholic and Jewish faiths [19]. These hesitations are not uniform across all religious sects and vary greatly in context and practice. Interestingly, despite these perceptions, studies comparing religious with non-religious communities in select African countries reported higher vaccination coverage for children raised in Christian and Muslim families than children from families without religion [20]. Similarly, the complete immunization status of children aged 0 to 1-year-old was found to be higher in the Christian communities than in non-Christian ones [21].

In a time where vaccine hesitancy poses a significant challenge to public health, it is important to identify the intricate interplay of factors exemplified at the individual and societal levels which influence vaccination behaviours. This paper aims to address a pressing research gap by reviewing literature on the role of religiosity on vaccine hesitancy, acknowledging its significant influence on social behaviour. Additionally, it aims to examine how religiosity interacts with other sociodemographic indicators in the context of vaccine hesitancy. It asks, what is the role of religiosity on vaccine hesitancy and if/how research has considered the connections between religiosity and other sociodemographic indicators associated with vaccine hesitancy. Through this analysis, we aim to shed new light on the dynamics of vaccine hesitancy among religious groups, contributing to the broader effort to promote vaccine uptake, dispel misunderstandings, and encourage constructive dialogue with these groups. Our intention is not to critique or undermine any religious belief but to enhance our collective understanding of vaccine hesitancy, an imperative step in safeguarding public health.

## Methods

We used an abbreviated version of the PRISMA-ScR (Preferred Reporting Items for Systematic reviews and Meta-Analyses extension for Scoping Reviews) using the 20-point checklist to guide this review and improve methodological rigor [22]. The original objective was to scope literature analyzing the intersection of religious beliefs on vaccine hesitancy on a population level.

### Search & screening strategy

**Fig. 1:**
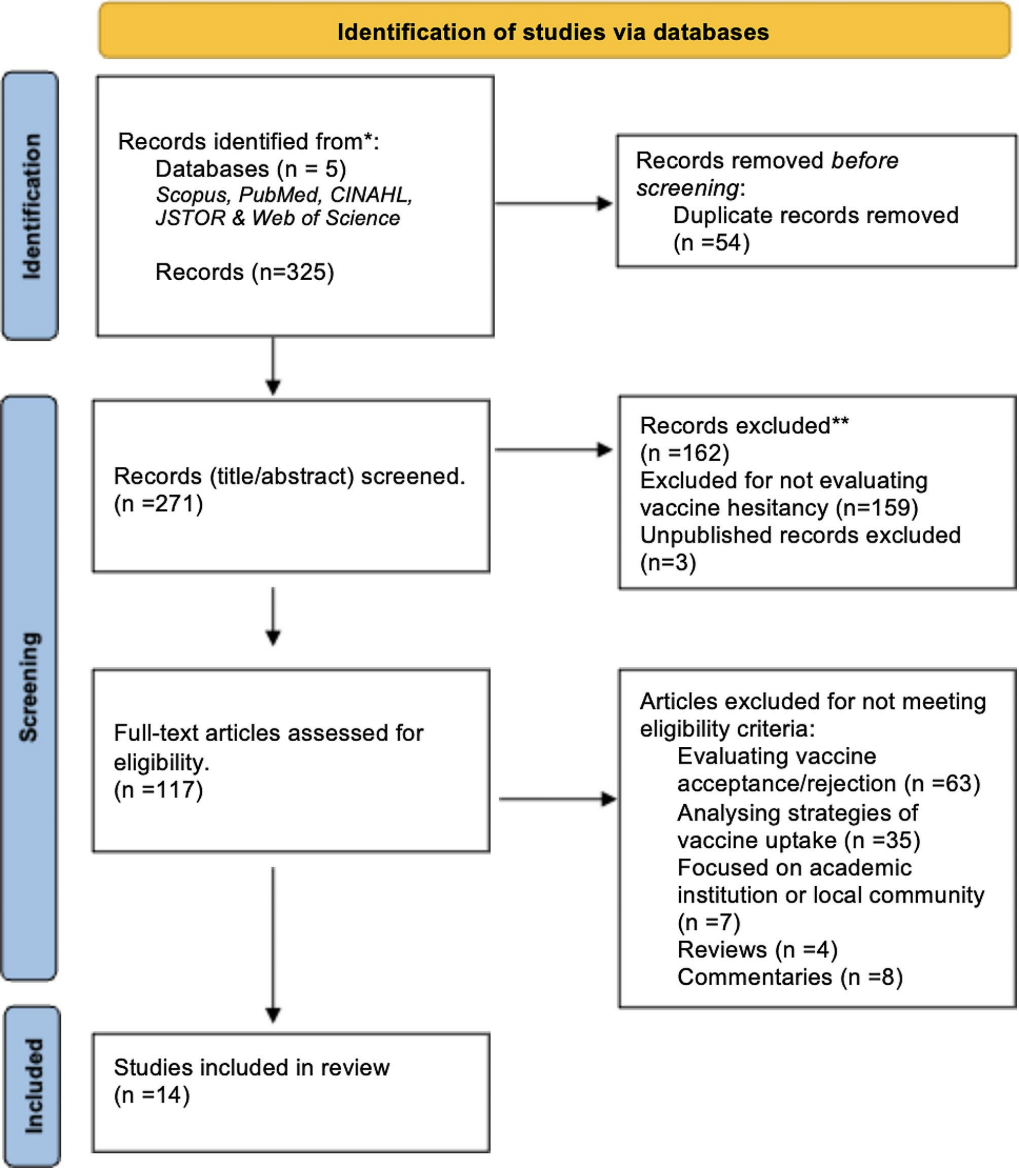
PRISMA (Preferred Reporting Items for Systematic Reviews and Meta-analyses) flow diagram

We searched and cross-referenced results from five databases (Scopus, PubMed, CINAHL, JSTOR, and Web of Science), using Boolean operators. We used key words such as (Vaccine hesitancy OR Immunization hesitancy), (Religious beliefs OR Religiosity OR Religious groups), (Outbreak OR Syndemic OR Epidemic OR Pandemic), (Population OR Country)( See Fig. 1). The inclusion criteria for our study were that the literature should be: (1) written in English, (2) concerned with vaccine hesitancy as the focus of study, (3) study the impact religiosity or religious beliefs as either an outcome or control variable, 5) population level, 6) peer-reviewed articles. No limits were placed on geography, type of vaccine or infectious disease, nor publication date. Excluded literature from our study were those not focused on population levels, such as academic institutions, localized communities etc. Additionally, experimental design studies or general articles regarding vaccines, with no mention of religion/religiosity. We did not include articles which focused only on vaccine acceptance or rejection, nor those which focused on strategies to improve vaccine uptake without discussing hesitancy specifically. We only included literature which presented original findings, excluding reviews and commentaries. As we only included peer-reviewed articles, we did not assess quality assuming the review process ensures a degree of quality assurance.

### Data extraction

All identified studies were imported into the systematic review software Covidence (Veritas Health Innovation Ltd), where duplicate studies were automatically removed. A single reviewer screened titles and abstracts, then full texts, for inclusion. Once all identified articles were screened, a custom data extraction form was developed within Covidence. A single reviewer extracted data pertaining to the following: author, title, abstract, year of publication, religious identity, study design, methodology and assessment tool used, geographical location of study, infectious disease or vaccine used, social identifiers of participants (if identified), impacts of religious beliefs, and key findings. A thematic analysis was utilized [23], providing an organized portrait of the data, with articles categorized based on common findings.

### Study limitations

Inclusion of only peer-reviewed literature did mitigate the risk of bias to a degree [24]. However, the exclusion of grey literature disregards learnings from government or civil society reports and media articles that could have proved useful to understand different aspects and impacts of vaccine hesitancy on varying populations. Including articles available only in English also reduced the breadth of literature, likely limiting geographical focus.

## Results

### Description of articles

We analysed 14 peer-reviewed articles that included components related to religiosity or religious beliefs and their impact on vaccine hesitancy published until September 2023 (when we completed the search) [see Additional file 1]. All the articles were published in approximately the last decade between 2012 and 2023, with only 4 of the articles published before 2020 (Table 1). The research presented in the articles was primarily conducted in the Unites States (42.8%) and the Netherlands (14.2%), with single studies in the remaining countries. Only 1 country of study was found from Africa, 2 from Southeast Asia, 1 from Oceania, and the rest from western countries (Table 2). Among the ones published before the COVID-19 pandemic, only 2 articles were focused on hesitancy regarding a specific vaccine, the Oral Polio Virus (OPV) vaccine and Measles, Mumps, Rubella (MMR) vaccine respectively with the remaining studying general vaccine hesitancy (Table 3).

**Table 1:** Breakdown of articles by Publication Year (*n* = 14)

Year	*n* (%)
2012	2 (14.2)
2017	1 (7.1)
2018	1 (7.1)
2020	2 (14.2)
2021	1 (7.1)
2022	5 (35.7)
2023	2 (14.2)

**Table 2:** Breakdown of articles by country the study was conducted in (*n* = 14)

Region	Country	*n* (%)
North America	United States	6 (42.8)
Europe	Netherlands	2 (14.2)
Europe	Poland	1 (7.1)
North America	Canada	1 (7.1)
Oceania	Australia	1 (7.1)
Southeast Asia	Indonesia	1 (7.1)
Southeast Asia	Malaysia	1 (7.1)

**Table 3:** Breakdown of articles by vaccine of study conducted (*n* = 14)

Vaccine	*n* (%)
COVID-19	9 (64.2)
OPV	1 (7.1)
MMR	1 (7.1)
General	3 (21.4)

Out of the 14 studies included in our review, twelve utilized quantitative methods, while the remaining two employed qualitative approaches. Quantitative studies predominantly relied on surveys, with respondents numbering anywhere from 236 to 23,106. Many of these studies made use of established questionnaires, including the Anti-vaccination scale, Santa Clara Strength of Religious Faith, the Vaccine Hesitancy scale, and variations thereof [25, 26, 28, 36]. The studies used a combination of questionnaires to answer varied research questions [27, 29, 31–34, 37, 38]. Nearly all studies conducted data collection online, except for one [32]. Among our pool of articles, three of the studies relied on national population surveys [31, 33, 37] with the remaining conducting their own cross-sectional analysis with convenience sampling [25–29, 32, 34, 36, 38]. Among the studies employing national population surveys as the data collection tool, two studies used time-trend analysis across a decade to show population intentions [31, 37]. The two qualitative studies gathered data through focus groups and semi-structured interviews respectively, involving participant numbers ranging from 27 to 52 [30, 35].

Among the studies included in our analysis, we found various approaches to categorizing religious belief and identity. Five articles did not explicitly specify religious beliefs or identities but rather assessed them homogenously within the study using a binary (Y/N) categorization. In two articles respectively, the focus was on hesitancy among Muslim and Christian populations without further stratification within these groups. In another single study, however, Christians were further categorized into subgroups such as Evangelical, Protestant, and Catholic. Additionally, three studies examined multiple religious groups (Christians, Muslims, Hindu, Jews etc.) within their sample populations (Table 4).

**Table 4:** Breakdown of articles by religious groups studied (*n* = 14)

Religious groups	*n* (%)
None specified	5 (35.7)
Muslim	4 (28.5)
Christianity (un-specified)	2 (14.2)
Catholic	3 (21.4)
Protestant	3 (21.4)
Others (including Jews, Baptist, Hindu)	1 (7.1)

### Impact of religiosity

In a simplified approach with religious beliefs as the sole identifier for hesitancy, it was found to quite often significantly and negatively associate with the intention to vaccinate [26–29]. Faith was seen as a strong influence on an individual’s decision to vaccinate with religious individuals relying on their faith rather than seeking vaccination [26, 27]. It is important to acknowledge that faith can play both positive and negative roles with the intention to vaccinate. Some studies indicate religious beliefs could be seen as conflicting with vaccination recommendations, viewing illnesses and diseases as something that happens because of fate or divine will [27]. One qualitative study shed light on how religious communities perceived the regional government’s communication, which they felt were disparaging their choices. Participants expressed that such actions ‘put an ill connotation on it for a lot of people [who say] this politician is making fun and singling us out’ [35]. Another qualitative study, exploring the thinking behind the decisions for people to vaccinate themselves and their children, found that decision-making was linked to participants’ trust in God and fear of regretting decisions on vaccination in the future. Contrastingly, another group of parents within the same population saw vaccines as “a gift from God to be used in gratitude” [30]. Vaccination attitudes were observed to be linked to the prospect of having children, showing a negative correlation with being religious [28]. However, a contrasting observation was made within a time-lapse analysis, where religion was not associated with vaccination decision-making [31]. Similarly, another study in Nigeria found that the role of religion played no influence with the propensity to refuse the OPV vaccine [32]. The findings from these studies saw that across similar communities with reflecting religious observations, other determinants such as education and socioeconomic status played more substantive pivotal roles towards their attitudes to vaccinate [31, 32].

Four studies examined the influence of the Christian faith on vaccine hesitancy. All these studies independently found a statistically significant, negative association between vaccine hesitancy and religiosity [30, 33–35]. The role of religious doctrine in this association with vaccines was frequently mentioned. Wilhelmina et al. captured this sentiment in their interviews, with one participant: “I know for sure that God cares for me. And that the things He sends me, that may also be disease, that He will help me cope with it.” Their belief system was pillared on the premise that “Men should not interfere with divine providence because God is almighty” [30]. The belief in God or a higher power’s ability to intervene in the world proved to be a more prominent predictor of vaccine intention compared to a simple belief in God or a higher power. Specifically, a one unit increase in the belief in the intervening power of God, or a higher power was associated with an approximately 62% lower intention to receive a COVID-19 vaccine [34]. This finding may help explain why Catholics were observed to have significantly higher odds of being vaccinated than evangelical Protestants [34]. Additionally, religious belief has been found to create cognitive dissonance, causing individuals to adhere to their narrative despite being faced with evidence to the contrary [35].

The studies which focused on Muslim or Islamic communities found that adherence to the Islamic faith had a considerable influence on hesitancy to vaccinate. Harapan et al. observed a noted increase in vaccination coverage among Muslims, rising from 49 to 82% between 1991 and 2012. However, this increase was followed by an increase in vaccine hesitancy among the populace in later years, coinciding with the introduction of fatwa—an edict by a local governing religious authority, though not legally binding but highly influential among the Muslim population. Their ruling, in 2017, stated that immunizations were allowed but the vaccine should be designated as ‘halal’ by their governing body. The perception of the use of porcine ingredients in the manufacturing process of the MMR vaccine led them to reject this vaccine. This subsequently led to a marked decline in coverage [37]. It is challenging though to establish clear links between this introduction of the fatwa and its impact on individuals’ vaccination intentions without further research. This difficulty is underscored by the observation that a province with a distinctly different religious body also showed lower vaccination coverage than others [37]. Another study that specifically examined religious beliefs found that, similar to Christians, Muslims were also significantly influenced by their perception of a higher power controlling health and illness, which was associated with lower intent to receive a COVID-19 vaccination [38]. Such beliefs fed into the mindset that death is inevitable and thus interventions such as these were futile [38].

Reviewed studies document variations in vaccination hesitancy among different religious groups. Notably, vaccine hesitancy was comparatively higher among evangelical Christians when compared to Catholics, other Protestants and Muslim groups. Possible explanations mentioned in the study include lack of trust in science, the belief in divine healing and protection from God, and conspiracy beliefs about vaccines [25]. Those with no religious affiliation were also found to have amongst the highest vaccine hesitancy levels [25]. This group may reflect those with historical and cultural contexts which is unrelated to their religious beliefs, perpetuating racial disparities, and those most a risk of misinformation regarding viruses and vaccines [25]. The Mollema et al. study, on the other hand, found that individuals without religious beliefs were less like to hesitate to vaccinate [31]. While smaller religious sects, such as Reformed Congregations and Reformed Bonders, participated less in vaccination programs than other religious observants [31]. These sects’ belief that such interventions deviate from Gods plan contribute to their hesitations.

### Intersections between religion and other social factors

We further asked if research on religion/religiosity and vaccine hesitance included other social demographic factors in the analysis, recognizing religion as only one of many factors that might influence attitudes towards vaccination. All studies in our review reported sociodemographic factors to some degree related to vaccine hesitancy within their sample populations. The frequently assessed sociodemographics were gender, education, and socioeconomic status or income. The lesser used constructs included age, family composition, race/ethnicity, political affiliation, and, in one paper intensity of religious observation. Notably, income, education, and age were found to be significantly and positively associated with the intention and willingness to vaccinate [29, 31, 33, 36, 37]. Age exhibited a U-shaped relationship with vaccine hesitancy, being lower in younger individuals, increasing significantly with age until it reached a peak in middle-aged groups, and then declining in seniors [31, 33, 36]. In our set of articles, gender was examined considering men and women as the main categories. However, it is worth noting that an ‘other’ category was included in most surveys but consistently yielded statistically insignificant results and so was removed from the final analysis. A study by Olagoke et al. assessed sex, male or female, instead of gender and found no significant difference between the sexes [27]. In six studies, Women were found to have higher levels of vaccine hesitancy than men [25, 26, 32, 34, 35, 38]. Being married and having children was associated with even higher levels of vaccine hesitancy than those who had never married or had children [28, 31, 32]. In some instances, however, men with children exhibited, especially regarding their first born, higher levels of vaccine hesitancy in comparison with women [28, 30]. Education was found to have an inverse relationship with vaccine hesitancy, with such attitudes decreasing as education levels increase. Better grasp of the medical and scientific benefits of vaccines aided to make this improvement [25, 26, 28, 33–37]. A similar trend was noted for income or socioeconomic status [25, 26, 28, 33], but a population level survey analysis in one study, found a complementary relationship between income, religion, and vaccine hesitancy [37]. Though access in terms of time cost and convenience to immunization center is considered an underlying factor for this difference [37].

The impacts of political affiliation were analyzed in three out of five of the studies conducted in the United States. During the recent COVID-19 pandemic, political affiliation has been observed to play a significant role in vaccine-related decisions [25, 34, 36]. It may be attributed to shifts in social norms among right-wing conservatives and news outlets [25]. The DiGregorio et al. study identified a strong association between political affiliation and the intention to hesitate when it comes to vaccinations [34]. Notably, flu vaccination intentions were found comparable among the 3 major political groups: Independent, Democrats and Republican [25]. One study found Republican political identification to be correlated with a higher likelihood of receiving all (COVID-19 & flu) vaccinations [25]. Interestingly, when controlled for age, Republican affiliation was associated with higher vaccine hesitancy and higher odds of rejecting the COVID-19 vaccine [25, 36]. Additionally, Independents more than Democrats, were found to exhibit vaccine hesitancy during the COVID-19 pandemic [25, 36].

Our review found four articles that elaborate on the impact of race on vaccine hesitancy. Racial minorities tend to exhibit significant higher levels of vaccine hesitancy [25, 27, 34, 36]. This hesitancy varies across the racial identities. African Americans and Hispanics were found to have the highest levels of vaccine hesitancies, ranging from 48 to 60%, in comparison with other racial identities [25]. Furthermore, these two racial identities had relatively lower levels of trust in information from scientists about the virus. While White and Asians within this sample had higher levels of trust [36]. This may account for higher odds of vaccination among Asian, White, and non-Hispanic individuals within the population sample [34]. Scientific information was trusted by the vast majority within these communities indicating that how information is shared is perhaps more important than the actual content [36].

Amongst our articles, half of them studied the intersections of these sociodemographic with religious beliefs. These studies employed multivariate analysis to study the impact of intersecting social identities relating to vaccine hesitancy. Most of the included studies showed that when analysed with other sociodemographic factors in multivariate analysis, religious beliefs were found to be no longer significant [29, 31, 33, 34, 36, 38]. For example, African Americans with reported levels of religiosity were likely to exhibit much higher rates of COVID-19 related vaccine hesitancy than White Americans or Asian Americans [36]. Likewise, lower income and educational attainment, in conjunction with non-Western heritage, played varying yet influential roles in determining the choice to participate in endemic and voluntary enrollment within ongoing vaccination initiatives [31]. These studies found that the social, historical, and contextual factors (present and past) played a larger role with the decision to vaccinate than did religious convictions or beliefs. Trust in public or healthcare institutions or perceived relationship between community and governing agencies were seen as more consistent indicators of willingness to access vaccination [28, 32]. One study did not reflect the above findings. This outlier study found that religious beliefs continued to play role in vaccine hesitancy particularly within the higher socioeconomic bracket among Muslims [37]. It found that adherence to religious traditions, impacted perceptions of vaccines especially with faith politics becoming more popular in the current political climate [37].

## Discussion

Vaccine hesitancy remains a challenge that compromises the success of vaccination efforts and the safeguarding of the population against diseases [39, 40]. A previous study employing the matrix of determinants of vaccine hesitancy developed by the SAGE Working Group found the three most reported reasons for vaccine hesitancy are: vaccine related concerns, knowledge, and awareness issues, and thirdly religion [41]. Religion serves as a major unifying force in the lives of billions worldwide, and it has the potential to shape individuals’ perspectives. Neglecting this connection or isolating science and religion as entirely distinct realms could hinder our ability to comprehend and influence people’s sentiments regarding scientific matters [40]. In this review, we analysed the influence of religiosity on vaccine hesitancy, assessing how religion influences attitudes towards and decisions around vaccine hesitancy, both independently and in conjunction with other sociodemographic factors.

In most studies when religion is uniformly regarded as the sole determinant of vaccine hesitancy, it consistently emerges as a significant factor in contributing to vaccine hesitancy. The identification of religious beliefs was shown to be associated with higher levels of vaccine hesitancy than those with no religious affiliation [26–29]. This was in line with earlier findings from the WHO working group [41]. However, some studies that did not reach a similar conclusion emphasized that other sociodemographic factors were more common indicators of vaccine hesitancy than religion alone [31, 32]. More research is needed about the degree to which religion influences attitudes towards vaccination in relation to other factors.

While eleven of the studies primarily examined secular western countries, it’s worth noting that the regions with the highest rates of religious affiliation (approximately 95% or more) are in Africa, South Asia, Central Asia, and the Middle East. Furthermore, the regions with the highest rates of people reporting that their religious teachings and science disagree are Northern America and Southern Europe, where a smaller proportion of the population identifies with a specific religion [42]. This is also reflective in our findings. The only exception is a single study conducted in the Netherlands, characterized by a predominantly non-religious populace among individuals aged 15 and over, which may help explain this distinctive result [43].

The review identified just one article that comprehensively assessed the extent of religious observance within religious communities [32]. It is not surprising to find this scarcity, with only 14 studies at hand; however, it underscores a noticeable gap in the existing literature. This measurement of religious commitment has been found to effect individuals’ behavioural adherence to religious values, beliefs, and practices [44], and thus further research is warranted.

Nonetheless, our findings did emphasize that vaccine coverage varies across different religious groups, underscoring the contextual factors that contribute to vaccine hesitancy within these groups. While some similarities were observed among diverse religious groups, such as a shared belief in divine healing and protection from God, other reasons for hesitancy included strict adherence to religious doctrines and concerns about potential regret related to vaccination decisions. The limited literature addressing the distinctions between various religious identities result in generalizations of ideological beliefs being applied to the entire population.

Our findings on sociodemographic factors align with a recent scoping review that investigated the influence of such factors on COVID-19 vaccine hesitancy [45]. When considering such factor, e.g., gender, race, education, religious beliefs were found to be no longer a significant indicator within a population for vaccine hesitancy [29, 31, 33, 34, 36, 38]. This may be explained by disparities in access to and confidence in vaccines among traditionally marginalized communities such as BIPOC, LGBTQ+, and immigrant communities [46–48]. The structural oppression that these communities have and continue to face contribute to prevailing health inequities and growing mistrust of medicine and governmental institutions [47]. However, other research focusing on infectious diseases, have found that, it is the complex interplay of intersecting social identities that matters. Individuals’ actions and attitudes are influenced by various social identities, encompassing race, religion, and class and others [49]. Limiting analysis or scope to a single aspect of social position or demography at a time can impede our ability to fully grasp the extent of the disease’s social and health impacts, and its unequal distribution across diverse social groups within society [50].

This review presents implications to researchers and policy makers for understanding and improving population specific vaccination rates. Our analysis underscored the need for a nuanced approach to addressing vaccine hesitancy among religious groups. A comprehensive examination of vaccine hesitancy within religious groups entails investigating various factors such as secular, social, and ecological elements, in addition to concerns about long-term side effects. Recognizing these aspects is crucial, considering both current and historical structures that shape their inequities. This recognition helps to understand their contributions to the intricate landscape that shapes vaccination intent and behavior within these populations [29, 34, 36]. Gender and family considerations, for example, could inform engagement strategies and policies by recognizing the higher tendencies of women with children to be hesitant in vaccinating themselves and families [28, 32]. Additionally, initial targeted interventions for those living in lower income or socioeconomic status religious neighbourhoods could aid to address disinformation before trust in government is eroded [29, 31, 33]. Furthermore, the observed significant differences in belief in God or a higher power between Catholics and Protestants during the COVID-19 pandemic suggest the importance of tailoring targeted policies to specific religious communities. This insight highlights the potential for developing interventions that resonate with the diverse beliefs present within religious groups, ultimately addressing the inconsistencies in vaccine acceptance [30, 34]. The literature strongly advocates for cultivating strong connections with religious leaders, recognizing their influential role in shaping opinions and beliefs. Endorsements from these leaders can serve to solidify confidence in vaccine safety, making them crucial partners in public health efforts [27, 37, 38].

This review emphasizes the necessity for additional research into the relationship between vaccine hesitancy and religious influences. It highlights the need for researchers to identify, develop, and disseminate effective strategies for vaccine distribution aimed at achieving equitable coverage. By focusing on this gap in knowledge, researchers can advance our understanding of vaccine acceptance and develop targeted interventions to address challenges.

Certain limitations were noted for this review as the scope was limited to peer-reviewed publications, completed by a single reviewer, and did not search the grey literature. As such, it is important to note that this review is not exhaustive, and other evidence may exist outside of peer reviewed scientific literature that could have contributed to the findings and may have been inadvertently excluded. Secondly, this review was limited to English language articles, as such, future efforts should expand this investigation to non-English journals to improve the generalizability of results. Furthermore, our search strategy did not include articles on closely related topics such as vaccine acceptance, rejection, or vaccination strategies; further research on these topics, particularly understanding the impact of intersecting identities, is also needed. Lastly, the religions included and analysed in our review is limited to the literature available, and thus are limited in its scope to portray vaccination intentions for the many religious identities that are not considered.

## Conclusion

Vaccine hesitancy is a complex issue and driven by a myriad of individual and societal factors among which religious beliefs is commonly associated to be a driver of higher levels among populations. The challenge of vaccine hesitancy looms large, affecting the success of vaccination campaigns and, in turn, the safeguarding of populations against preventable diseases. Understanding the context of religion in the lives of community members is crucial in order to comprehensively evaluate its role in shaping vaccine attitudes and behaviors. This review calls attention to this intricate relationship. It underscores the need for nuanced, culturally sensitive strategies to address hesitancy within religious communities, recognizing the impact that social factors play, and advocates for further intersectional research to better understand and combat this critical public health challenge.

### Electronic supplementary material

Below is the link to the electronic supplementary material.

**Additional file 1**: Summary of included studies
It is a descriptive table highlighting the 14 studies discussed in the article. The table identifies the vaccine of focus, religion studied and the key findings for each article

## Data Availability

No datasets were generated or analysed during the current study.
