# Supplementary material for: Faith and vaccination: a scoping review of the relationships between religious beliefs and vaccine hesitancy
Source: BMC Public Health. 2024 Jul 6;24:1806. doi: 10.1186/s12889-024-18873-4 (PMC11227154; doi:10.1186/s12889-024-18873-4)
Supplement: Supplementary file 1 — Additional file 1: Summary of included studies It is a descriptive table highlighting the 14 studies discussed in the article. The table identifies the vaccine of focus, religion studied and the key findings for each article [file 12889_2024_18873_MOESM1_ESM.docx]

Additional file 1.

**Summary of Included Studies**

| Articles | Vaccine of focus | Religions studied | Key Findings |
| --- | --- | --- | --- |
| Zhang et al. 2023 | COVID-19 | Catholicism, Protestantism, Islam | - By religion, vaccine hesitancy was relatively high among Evangelical Christians (46%) and those with no religious affiliation (42%), and it was low among Catholic/Orthodox (32%), Mainline Protestant (27%), and Muslim (34%) groups. - Vaccine hesitancy was relatively high among Black (60%) and Hispanic (48%) participants compared to Non-Hispanic White Americans (35%). - Vaccine hesitancy was relatively high among Independents (45%) and Republicans (47%), with Independents having higher proportions of not having received a vaccine (27% for COVID-19; 51% for flu). |
| Taylor et al. 2017 | Oral Poliovirus (OPV) | Islam, Christianity | - Individual household religious and ethnic identity, as well as a composite index of households' intensity of religious observation, did not strongly influence the propensity to refuse OPV. - Little evidence was found that ethnicity, religious identity, or measures of religious observation were directly associated with the propensity to refuse OPV. Settlements with very similar ethnic and religious profiles exhibited varying levels of refusal risk. Moreover, at the household level, our index of the intensity of religious observation was not significantly associated with the propensity to refuse OPV. |
| Olivera-Figueroa et al. 2023 | COVID-19 | Christianity | - A strong influence of faith on one's decisions reduced vaccination intention. - Past Negative enhanced vaccination intention, whereas Past Positive reduced vaccination intention. |
| Rozbroj et al. 2019 | General | Christianity | - Negative vaccine attitudes were significantly associated with being male, having children, being religious, and not voting for major political parties. |
| Wong et al. 2022 | COVID-19 | Islam | - A high acceptance of the COVID-19 vaccine among the Muslim population, with 57% expressing a definite intent to get vaccinated. - Younger age and higher income were associated with a higher likelihood of having a definite intent to be vaccinated against COVID-19. - significant proportion of participants indicated they would not take the vaccine unless it was certified halal, while a majority reported willingness to take the vaccine in emergency situations where no alternative medicine is available. |
| Jacobi et al. 2021 | COVID-19 | Catholic, Jewish, Baptist, Evangelical, Mormon, Hindu | - Across all races, there is high trust in COVID-19 information from scientific experts (82–90%). Most respondents, predominantly female, with high household incomes, and educated at a degree level or higher, show an average age of around 58 years. - African Americans exhibit the lowest anticipated COVID-19 vaccine acceptance compared to Whites, with stark disparities in odds ratios (OR 0.19, 95% CI 0.12–0.29). While Hispanics also show lower acceptance (OR 0.38, 95% CI 0.24–0.62), Asian Americans and other racial groups do not significantly differ from Whites. - Worry of the detrimental health effects of COVID-19 plays a significant role in shaping vaccine attitudes, becoming a significant factor in full models (OR 1.54, 95% CI 1.05–2.26). - With expanded efforts to build trust, understanding, and collaboration in religious settings, particularly focusing on racial minorities, there is an opportunity to mitigate the detrimental effects of the ongoing pandemic within these communities. |
| Harapan et al. 2021 | Measles, Diphtheria tetanus toxoid & pertussis (DTP3) | Islam, Christianity | - Between 1991 and 1997.there has been a substantial reduction in vaccination coverage disparities across wealth index quintiles. Vaccination coverage increased steadily from 40% to 73% in the poorest quintile and from 70% to 84% in the richest quintile. - There was an increase in measles vaccination coverage among both non-Muslims (from 57% to 79%) and Muslims (from 59% to 79%). However, there were fluctuations in coverage over time, with stagnation observed among Muslims between 2012 (81%) and 2017 (79%). - While non-Muslims initially had higher vaccination coverage in 1991, the disparity decreased gradually over time. However, there were fluctuations, possibly influenced by factors such as the issuance of fatwas and variations in vaccination coverage by province, with Aceh, where Shariah Law is practiced, having the lowest measles vaccination coverage at 54% in 2017. |
| DiGregorio et al. 2022 | COVID-19 | Christianity | - Income, age, education level, and being a Twitter user are positively associated with the likelihood of having received or planning to receive a COVID-19 vaccination. Conversely, political affiliation, regional location (e.g., West South Central), and identification as "Other, non-Hispanic" are negatively associated with vaccination intent. - Asian, non-Hispanic individuals have higher odds of vaccination compared to White, non-Hispanic individuals, while those identifying as "Other, non-Hispanic" are less likely to be vaccinated. Additionally, Catholics have higher odds of vaccination compared to evangelical Protestants, and Christian nationalism is negatively associated with vaccination intent. - Belief in a God or higher power that intervenes in the world is negatively associated with the likelihood of being vaccinated against COVID-19. Conversely, belief in a supervising God or higher power does not significantly impact vaccination intentions. Additionally, religious service attendance is positively associated with vaccination intent, suggesting a complex relationship between religious beliefs and vaccination behavior. |
| Capurro et al. 2022 | COVID-19 | Christianity | - The main drivers identified were concerns about vaccine safety, religious and conservative views, and distrust in government and science. Participants expressed uncertainty about the novel COVID-19 vaccines and potential long-term side effects. Safety concerns and distrust in government decisions, such as administering the AstraZeneca vaccine despite international skepticism, contributed to vaccine hesitancy. - Religious and conservative beliefs played a significant role in vaccine hesitancy, with mention of cognitive dissonance among religious individuals who deny the reality of COVID-19 despite personal experiences. |
| Mollema et al. 2012 | General | Catholicism, Protestantism, Reformed Bonders, Reformed Congregations | - Individuals aged 10-29 years, those with low income or educational levels, non-Western descent, and members of specific religious groups or with anthroposophic beliefs regarding vaccination were less likely to participate in vaccination programs. - Lower participation in the National Immunization Program (NIP) among those with low educational levels or income is consistent with observations that areas with low socioeconomic status tend to have lower uptake of vaccinations, - Members of Reformed Congregations, Reformed Bonders, and those with anthroposophic beliefs participated less in the NIP compared to those with no religion or different beliefs. However, no associations were found for religion, income, and educational level with parents' intentions to accept remaining vaccinations for their child, contrasting with findings for NIP participation. |
| Maciuszek et al. 2023 | COVID-19 | Christianity | - Religiosity was higher among undecided individuals compared to those willing to vaccinate. However, there were no significant differences in religiosity between the unwilling-to-vaccinate group and either the undecided or willing-to-vaccinate groups. - In the unwilling-to-vaccinate group, higher religiosity was associated with lower trust in science. In the undecided group, higher conspiratorial thinking was associated with higher trust in science, while higher religiosity was linked to lower trust in science. In the willing-to-vaccinate group, higher conspiratorial thinking was associated with higher religiosity, and higher religiosity was associated with lower trust in science. However, there was no significant correlation between trust in science and conspiratorial thinking in this group. Additionally, religiosity was higher among undecided individuals compared to those who had been vaccinated. - Higher levels of conspiratorial thinking were associated with higher religiosity, while higher trust in science was linked to lower religiosity. Additionally, trust in science was weakly associated with lower conspiratorial thinking. |
| Levin et al. 2022 | COVID-19 | Christianity | - Statistical analysis showed that political variables remained significantly associated with skepticism even after adjusting for sociodemographic factors. However, for vaccine hesitancy, only political indicators remained statistically significant, while religious indicators did not. - Given the persistence of skepticism and hesitancy, alternative solutions are suggested. These may include broader government mandates, despite facing political resistance in some instances. Failing to address vaccine hesitancy could lead to the emergence of new variants, increased fatalities, and continued strain on the healthcare system. |
| Olagoke et al. 2021 | COVID-19 | Christianity | - Significant negative association between religiosity and intention to vaccinate against COVID-19, aligning with the concept of religious coping, which perceives crises as Acts of God beyond human control. - Religious leaders should be encouraged to educate their members on taking responsibility for their health, leveraging scriptural teachings emphasizing individual agency. Additionally, scientists could be urged to collaborate with religious institutions and leaders to foster trust and promote vaccine acceptance among followers. |
| Ruijs et al. 2012 | General | Protestantism | - Two distinct subgroups of parents emerged regarding the vaccination decision-making process: those who followed tradition and those who made deliberate choices. Traditional parents based their decision on religious doctrine, believing that interference with divine providence is inappropriate. In contrast, parents making deliberate decisions engaged in lengthy discussions and considered both vaccinating and not vaccinating. - Religious beliefs significantly influenced vaccination decisions for both groups of parents. While traditionally non-vaccinating parents cited religious doctrine as their primary reason for refusal, deliberately vaccinating parents viewed vaccination as a gift from God to be used in gratitude. Interestingly, traditionally vaccinating parents did not see any religious objections to vaccination and did not relate the decision to their belief in God. |
